# User Experiences of a Smartphone-Based Attentive Eating App and Their Association With Diet and Weight Loss Outcomes: Thematic and Exploratory Analyses From a Randomized Controlled Trial

**DOI:** 10.2196/16780

**Published:** 2020-10-02

**Authors:** Victoria Whitelock, Inge Kersbergen, Suzanne Higgs, Paul Aveyard, Jason CG Halford, Eric Robinson

**Affiliations:** 1 Department of Psychological Sciences University of Liverpool Liverpool United Kingdom; 2 Cancer Intelligence Cancer Research UK London United Kingdom; 3 School of Health and Related Research University of Sheffield Sheffield United Kingdom; 4 The School of Psychology University of Birmingham Birmingham United Kingdom; 5 Nuffield Department of Primary Care Health Services University of Oxford Oxford United Kingdom

**Keywords:** attentive eating, weight loss, smartphone app, eHealth, mHealth, food intake, obesity, overweight, focused attention, participant experience

## Abstract

**Background:**

Short-term laboratory studies suggest that eating attentively can reduce food intake. However, in a recent randomized controlled trial we found no evidence that using an attentive eating smartphone app outside of the laboratory had an effect on energy intake or weight loss over 8 weeks.

**Objective:**

This research examined trial participants’ experiences of using an attentive eating smartphone app and whether app usage was associated with energy intake and weight loss outcomes over 8 weeks.

**Methods:**

We conducted thematic analysis of semistructured interviews (N=38) among participants in the attentive eating smartphone app group of the trial who completed the 8-week assessment. Linear regression models examined the associations between energy intake and weight loss outcomes at 8 weeks and app usage.

**Results:**

Participants reported several barriers and facilitators to using the smartphone app, including repetition of app content, social setting, motivation, and habitual use of the app. Participants believed that using the app had some beneficial effects on their eating behavior and diet. Exploratory analyses indicated that more frequent recording of eating episodes in the app was associated with lower body weight (*B*=–0.02, *P*=.004) and greater self-reported energy intake (*B*=5.98, *P*=.01) at 8 weeks, but not body fat percentage or taste-test energy intake. Total audio clip plays, gallery views, and percentage of food entries recorded using an image were not significantly associated with energy intake or weight.

**Conclusions:**

Frequent recording of eating episodes in a smartphone app was associated with greater weight loss. There are barriers and facilitators to frequent use of an attentive eating smartphone app that may be useful to address when designing dietary behavior change smartphone apps.

**Trial Registration:**

ClinicalTrials.gov NCT03602001; https://clinicaltrials.gov/ct2/show/NCT03602001; Open Science Framework DOI 10.17605/osf.io/btzhw; https://osf.io/btzhw/

## Introduction

Lifestyle factors are the biggest contributors to disability-adjusted life years (DALYs) lost, with most DALYs lost attributable specifically to dietary factors (including excess body weight) having increased significantly from 2005 to 2015 [1]. In high-income English-speaking countries, obesity currently exceeds 30% in men and women [2]. Interventions are therefore needed that support people to change their diet and lose weight.

Smartphones are a viable way to deliver health interventions. For example, a large percentage (76%) of UK adults now own a smartphone [3], and smartphone apps can be used to deliver behavior change interventions [4]. However, sustained use of such apps may be needed for behavior change [5], as minimal usage could undermine effects of potentially successful interventions [4,6]. For example, Mattila and colleagues [5] found that sustained users of technology-based tools targeting a range of health behavior risk factors showed a greater reduction in body fat percentage and waist circumference than nonsustained users. Qualitative research has been used to explore factors associated with the use of smartphone apps designed to promote behavior change [7,8]. The results of such research can be used to identify reasons for poor or nonsustained use and can be used to inform future development of health behavior change smartphone apps. For example, the use of health behavior change apps has been found to depend on the app being user friendly, intuitive, accessible, and well structured, as well as having personalized features [7,9,10]. The ability to access novel and updated content and receiving feedback on progress toward goals have also been found to motivate app use [7,11].

Paying more attention to food being consumed, termed *attentive eating*, has been found to reduce snack intake 2-3 hours later in some [12-15], but not other [16-18], laboratory studies. If eating attentively is sustained over longer periods, it may support people in eating less and losing weight. We developed and tested the feasibility of a smartphone-based attentive eating app designed to encourage a more attentive eating style in daily life. In a feasibility trial, participants with overweight and obesity used the app and reported in qualitative interviews that they found the app easy and acceptable to use, and it increased their awareness of what they had been eating [19]. We subsequently conducted a randomized controlled trial testing the efficacy of the multicomponent, attentive eating smartphone app plus standard dietary advice, compared to standard dietary advice only, to support weight loss in adults with overweight and obesity [20]. We found no significant effect of using the attentive eating smartphone app, compared to the control condition, on weight loss (−0.10 kg, 95% CI −1.6 to 1.3) or energy intake after 8 weeks of usage. At the end of the trial period, semistructured interviews were conducted with participants who had used the attentive eating smartphone app in order to understand user experiences. Here we report the results of qualitative analyses of these interviews. These results showed that some participants experienced benefits of using the attentive eating app, which may suggest that specific patterns of app use may be associated with dietary change and weight loss. Therefore, we also conducted exploratory analyses to examine whether greater use of specific app functions was associated with greater reductions in energy intake and weight during the trial; the results are reported here.

## Methods

### Design and Sample

The trial was prospectively registered on the Open Science Framework [21] and retrospectively registered at ClinicalTrials.gov (NCT03602001). A full description of the trial design and methods are reported elsewhere [20]. In brief, this was an 8-week randomized controlled trial. Adults with overweight and obesity (mean BMI ≥25 kg/m^2^) were randomized to use a multicomponent attentive eating smartphone app, along with receipt of standard dietary advice delivered via a booklet and text messages once a week (intervention group), or to receive standard dietary advice only (control group). Standard dietary advice only for the control group was similar to previous studies [22]. Participants were also required to have no history of eating disorders or food allergies, self-reported by participant; be aged 18-65 years; be fluent English speakers; not be taking medication that affects appetite; not be pregnant; not be scheduled for weight loss surgery during the trial; own an Android or Apple smartphone (Android operating system versions 4.4-7.1; Apple operating systems iOS 8-10); and not currently be on a structured weight loss program or using other weight loss apps.

### Intervention Components

#### Standard Dietary Advice

All participants were provided with a booklet based on British Heart Foundation materials [23] containing tips on healthy eating (eg, the importance of a balanced diet, reducing calories and lower energy swaps, eating fruits and vegetables, reducing intake of high-fat and high-sugar foods and drinks, shopping, and eating out) and brief information about physical activity. Participants also received a text message once a week that repeated the information in the dietary advice booklet.

#### Attentive Eating App

**Figure 1:**
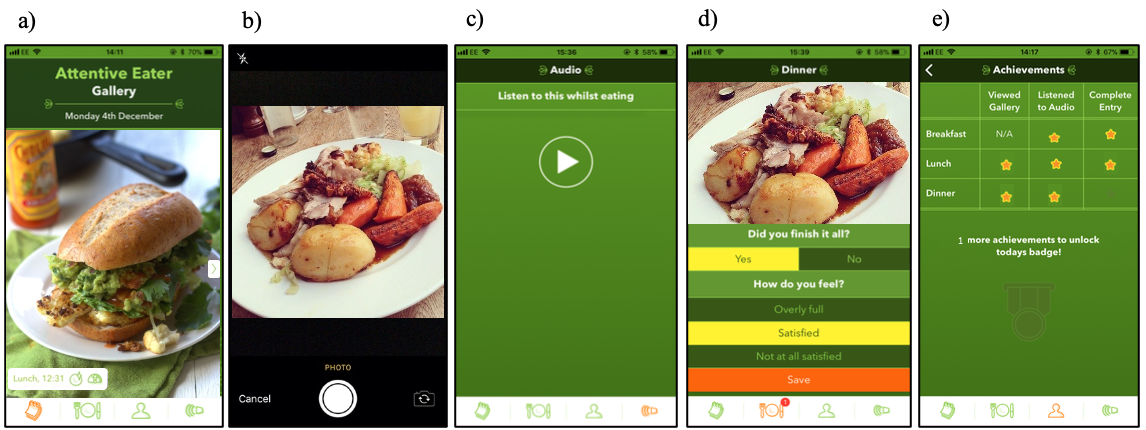
Screenshots of the key functions of the smartphone app: a) The food gallery; b) Photographing a meal; c) The attentive eating audio clip; d) Consumption experience questions; and e) Star and daily badge achievements.

The attentive eating smartphone app was designed to encourage a more attentive eating style by requiring users to photograph food and drink being consumed and then review this information when making dietary decisions throughout the day. Prior to eating and drinking, users accessed the camera function of their phones via the app and took a photograph of what they were about to consume. Alternatively, users could enter a description of the food or drink or upload a photo taken previously. Once they had finished eating and drinking, users answered questions about their consumption experience (ie, “Did you finish it all?” and “How do you feel?”). Once these questions had been answered, the food and drink entry was completed and the consumption episode, along with the photograph and answers to the consumption experience questions, were logged in a food gallery section of the app. Prior to deciding what and how much to eat for a meal, users of the app reviewed their consumption episodes for the day up to that point by navigating through the photographs and information about their consumption experiences stored in the food gallery. While eating and drinking, users were encouraged to listen to a 2.5-minute audio clip that prompted them to pay more attention to what they were eating. The audio clip did this by asking listeners to pay attention to the smell, taste, and texture of the food, as well as how full they felt. Users were also encouraged to eat slowly, one mouthful at a time, and to periodically think about how much food was on their plate at the beginning and how much they had eaten. To foster positive feedback, users received daily in-app *stars* for using the key functions of the app at appropriate times (eg, reviewing the gallery shortly before a meal) and received a daily badge if they obtained all of the stars in a day (see Figure 1 for screenshots of the app). Participants also received a short leaflet that explained the principles of attentive eating and other ways to eat attentively, such as avoiding eating while distracted.

### Outcomes

We used four measures collected at baseline and at the 8-week follow-up: (1) body weight, (2) body fat percentage (3), self-reported 24-hour energy intake, and (4) laboratory-measured bogus taste-test energy intake. Body weight and body fat percentage were measured with the Tanita BC-418 MA body composition analyzer (Tanita Corporation). Self-reported 24-hour energy intake was measured using myfood24, an online, automated 24-hour dietary assessment system developed and validated for use in the United Kingdom [24,25]. In the laboratory-measured bogus taste test, participants were provided with three bowls of three different kinds of biscuits, 50 g each—Maryland chocolate chip cookies, ~249 kcal; Cadbury’s chocolate fingers, ~240 kcal; and McVitie’s digestives, ~241 kcal—broken up into small pieces. In the laboratory, participants were given 10 minutes to rate the biscuits on 100-point visual analog scales, ranging from *not at all* to *extremely*, on a number of features (eg, crunchiness and flavorsome) and were told that they could eat as many biscuits as they wished [26]. Participants were asked not to eat for 1 hour prior to the assessment sessions in order to standardize hunger. Hunger was measured immediately before the taste test using a 100-point visual analog scale, ranging from *not at all* to *extremely*. The biscuits were weighed afterward in order to calculate the amount of biscuits consumed, and weights were converted to total kcals.

### Interviews

During the study visit at the 8-week follow-up, semistructured interviews were conducted with participants in the intervention group (ie, standard dietary advice and attentive eating app) to understand their experiences of using the attentive eating app. Participants were asked to be as honest as possible, were told that the researcher was interested in their experiences and opinions, and were told that there were no right or wrong answers. Participants were then asked what using the app was like for them and how they managed to include using the app into their lives. Participants were also asked what influenced how regularly they were able to use the app and whether using it affected awareness of what they were eating and/or what they ate. Interviews lasted approximately 10-30 minutes.

### Analyses

Semistructured interviews were audio recorded, transcribed verbatim, and imported into NVivo (QSR International) for analysis. Thematic analysis was conducted, following the steps of Braun and Clark [27]. The data were first coded using a systematic and inductive approach; initial codes were then aggregated into overarching themes by a single researcher (IK). The appropriateness of each theme and each code aggregated into a theme was then reviewed by a second researcher (VW). Any disagreements were resolved through discussion.

Ordinary least squares linear regressions were used to examine the relationship between app usage variables (ie, independent variables: total eating episodes recorded, total audio clip listens, total gallery views, and percentage of eating episodes recorded using an image) and each 8-week trial outcome (ie, dependent variables: body weight, body fat percentage, self-reported 24-hour energy intake, and taste-test energy intake), while controlling for baseline measurement of the dependent variables, total number of days in the trial, and hunger (in the analyses with taste-test energy intake as the dependent variable). The rationale for inclusion of independent variables was derived from the thematic analysis presented in the Summary of Thematic Analysis section. We analyzed the total number of food entries, audio plays, and gallery views, as participants were asked to complete these functions as often as possible. We analyzed the proportion of food entries that were photographed, as participants were asked to photograph as many of their meals as possible. A Bonferroni correction was used to account for multiple comparisons in statistical testing (0.05/4 regression models = 0.0125).

### Data Availability

The dataset for the quantitative analyses is available on the Open Science Framework [28]. The qualitative interview data are available upon request.

## Results

### Sample

Interviews were conducted with all 39 participants in the intervention group that attended the 8-week visit; however, due to equipment malfunction we were unable to transcribe one of the interviews, leaving 38 interviews. The exploratory quantitative analyses included all participants in the intervention group who completed all assessment sessions (N=39); see Table 1 for characteristics of the analyzed sample.

**Table 1:** Baseline characteristics of the analyzed sample.

Characteristic		Value (N=39), mean (SD) or n (%)
Age (years), mean (SD)		41.7 (10.3)
Gender (female), n (%)		31 (79)
Ethnicity (White), n (%)		35 (90)
**Education level, n(%)^a^**		
	Entry level or equivalent	0 (0)
	General Certificate of Secondary Education or equivalent	6 (15)
	Advanced (A) or Advanced Subsidiary (AS) level or equivalent	8 (21)
	Undergraduate degree or equivalent	15 (38)
	Higher degree or equivalent	7 (18)
	Other	3 (8)
Baseline BMI (kg/m^2^), mean (SD)		35.2 (7.2)
Baseline weight (kg), mean (SD)		99.5 (22.7)
Baseline body fat (%), mean (SD)		42.4 (8.5)
Baseline taste-test energy intake (kcal), mean (SD)		118.3 (98.4)
Baseline self-reported energy intake (kcal), mean (SD)		2047.0 (726.0)

^a^Percentages do not add up to 100 due to rounding.

### Summary of Thematic Analysis

Two overarching themes were identified: (1) barriers and facilitators to app usage and (2) effects of the app. See Table 2 for example quotes.

**Table 2:** Table of themes and supporting quotes.

Theme			Supporting quotes
**Theme 1: barriers and facilitators to app usage**			
	Subtheme 1: believing that the app was effective	PP^a^ 103: “I didn’t really see any effect of doing it so it just seemed like it wasn’t worth the effort.”	
	Subtheme 2: motivation to get the daily badge	PP 34: “It made me more aware to use the app rather than just like, ‘oh, I’ll just put that in later,’ it was like ‘no, I need to get my badge today.’” PP 64: “If you’ve messed up once in a day you tend to then think ‘well, I can’t get all the stars now today’ so you know you're not going to get the trophy. So you sort of don't feel quite as competitive about getting them, but if you get them from the beginning of the day, you think ‘right, I want to get them all today.’”	
	Subtheme 3: habitual, routine app use and distractions	PP 53: “I’d got into a routine that when I was in the kitchen or when I was doing meals the phone came, the picture came, everything came, but if I went out of the routine I was in, then I wouldn’t think to do it.”	
	Subtheme 4: not using full app in social situations	PP 53: “If I'm out socially with friends, family, or whatever and then to just kind of interrupt and say, ‘I have to listen to this for five minutes,’ it didn’t feel right to do it.” PP 35: “I think that’s a real age thing, I am not someone who posts my food on Instagram. Also feeling a bit embarrassed that I didn’t want to have to explain to people if they said, ‘why are you taking a picture of your dinner?’”	
	Subtheme 5: wanting a good-looking food gallery	PP 90: “I wanted it to look like a good gallery and not a bad one just for my own personal, I don't know, pride, satisfaction” and “it would make me want to eat more of those nice fresh homemade things.” PP 96: “I kind of stopped using it 'cause I was like ‘don't judge me.’”	
	Subtheme 6: meal descriptions used for retrospective entries	PP 64: “I ended up writing the description in more than the picture towards the end.” PP 03: “Where I’d forgotten to take a photo I was trying to write in the description. But I don’t think that that worked as well as it would have done if I had of taken a photo in terms of looking back over it.”	
	Subtheme 7: repetition of the audio clip	PP 103: “It was quite repetitive so I didn’t want to listen to the same thing over and over.”	
**Theme 2: effects of the app**			
	Subtheme 1: making healthier food choices	PP 92: “I had leeks last night on my carvery, which I never would have picked.”	
	Subtheme 2: eating smaller portions	PP 7: “I had rice and a sweetcorn dish last night, and normally I’d put two big spoons on, and last night it was just the one and the one spoon was plenty.” PP 71: “I bought a smaller plate; I said, ‘right that’s mine,’ so I am more aware of the portion size.”	
	Subtheme 3: reviewing the gallery informed eating	PP 78: “If I was hungry, I would look back to see what I've eaten, ‘ah yeah, you’ve ate plenty,’ and that would stop me.” PP 34: “I’ve noticed I’m very very bad at eating late at night.”	
	Subtheme 4: greater attention to hunger and fullness	PP 63: “I’ll stop because I’m full, and I’m not eating the full thing because I’m actually full halfway through it.”	
	Subtheme 5: eating more slowly	PP 4: “It’s helped me to slow down when I’m eating.”	
	Subtheme 6: eating attentively when not listening to the audio clip	PP 56: “Even though I’m not listening to it, I know what it asks me to do every time. So, yes, it would get ingrained in you and you would do it.” PP 98: “Even when I’m not out with the app, I definitely chew slower.”	
	Subtheme 7: enhanced eating experience	PP 22: “I noticed that I was tasting the food more and experiencing the food more.”	

^a^PP: participant; participant numbers exceed 39, as 104 participants (across arms) took part in the trial.

#### Barriers and Facilitators to App Usage

Key barriers to using the app included feeling that using the app in social settings was inappropriate, in particular, not wanting to be seen taking pictures of one’s food. We noted that older participants were more likely to describe these as barriers to using the app (see Table 2). Believing that the app was effective was identified as a motivator and facilitator to using the app, whereas appearing to not get anything out of using the app reduced motivation to use it. When a person was following their usual daily routine and had incorporated using the app into this routine, they found it easier to remember to use the app, while distractions reduced its use. Those who found the in-app stars and daily badge rewarding described these as motivators to use the app more frequently. However, missing out on a star achievement early in the day reduced motivation to continue using the app for the rest of the day. For some, wanting to have a good-looking diary motivated them to eat “healthier,” to eat nicer looking food, and to make sure they recorded this. For others, wanting to impress the researcher or feeling like their food gallery was going to be judged meant that they recorded food intake less often if they were going to eat food they considered incompatible with their goals. Being able to write descriptions of food consumed when someone had forgotten or was unable to take a photograph was helpful because it meant that participants could still complete their task even if they did not photograph the food. However, participants tended to take fewer photographs as time went on, despite finding it more difficult to remember how much they had eaten from the description than the photograph. Users found it boring to listen to the same audio clip repeatedly and this demotivated them to keep doing so.

#### Effects of the App

Participants reported that using the app helped them eat more slowly, pay more attention to hunger and fullness, make healthier food choices, and eat smaller portion sizes, and it enhanced the enjoyment of eating. Some participants described continuing to follow the focused attention principles described in the audio clip even when not listening to it during a meal, such as eating slowly. Participants also described using the food gallery to inform their eating choices and to increase awareness of their eating patterns.

### Exploratory Analyses

Usage of the app functions was generally reasonable: median total food entries was 136.0 (IQR 98), median total times listened to audio was 42.0 (IQR 66), and median percentage of food entries recorded using an image was 78.6% (IQR 28.1). However, median total gallery views (45.0, IQR 56) was low—the trial period lasted 56 days. VIF (variance inflation factor) (<0.96) and tolerance statistics (<4.33) showed that levels of multicollinearity were acceptable. There was no evidence that the number of audio clip plays, gallery views, or percentage of food entries recorded using an image were associated with taste-test or self-reported energy intake, weight, or body fat percentage at the 8-week follow-up (see Table 3). However, recording more food entries was associated with lower body weight and greater self-reported energy intake at 8 weeks. The number of entries recorded was not significantly associated with taste-test energy intake or body fat percentage.

**Table 3:** Regression results for app usage variables as predictors of trial outcomes at 8 weeks.

Outcome	Total food entries		Total audio plays		Total gallery views		Percentage of food entries using an image	
	*B* (95% CI)	*P* value	*B* (95% CI)	*P* value	*B* (95% CI)	*P* value	*B* (95% CI)	*P* value
Weight (kg)	–0.02 (–0.04 to –0.01)	.004	–0.01 (–0.03 to 0.01)	.46	0.04 (–0.002 to 0.08)	.06	0.01 (–0.03 to 0.04)	.66
Body fat (%)	–0.01 (–0.02 to –0.0007)	.05	0.004 (–0.01 to 0.02)	.62	0.01 (–0.02 to 0.04)	.50	0.002 (–0.02 to 0.03)	.84
Self-reported energy intake (kcal)	5.98 (1.50 to 10.46)	.01	–4.59 (–11.12 to 1.93)	.16	–7.55 (–20.49 to 5.38)	.24	4.50 (–5.88 to 14.89)	.38
Taste-test energy intake (kcal)	–0.06 (–0.58 to 0.46)	.82	–0.21 (–0.96 to 0.54)	.58	0.94 (–0.56 to 2.44)	.21	–0.19 (–1.37 to 0.99)	.74

### App Use Across the Trial Period

**Figure 2:**
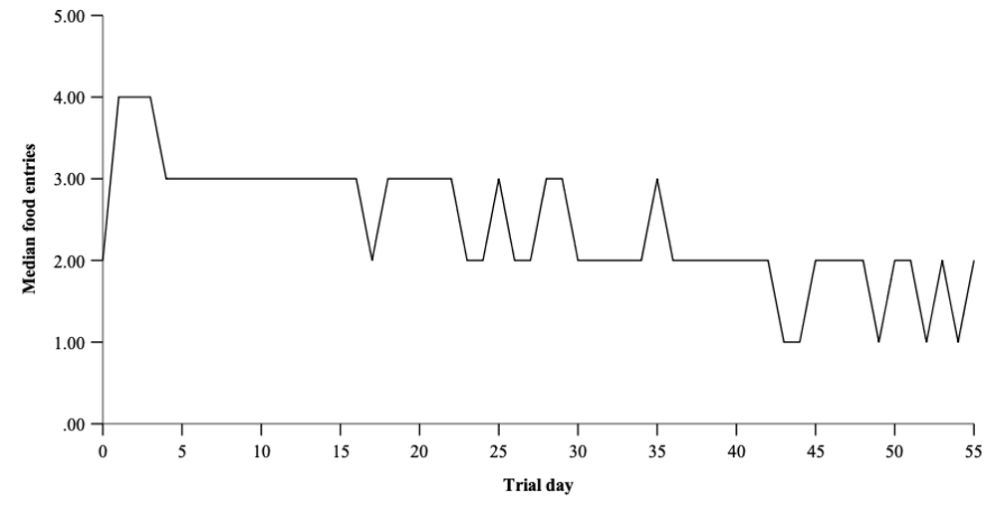
Median number of food entries across trial days for participants enrolled in the trial (0-55 days).

Total number of food entries was the only app usage variable significantly associated with any trial outcomes and was, therefore, plotted to explore usage over the trial period. The median number of food entries recorded per day declined over the trial period, with between three and four food items being recorded per day at the beginning of the trial, down to one to two per day toward the end of the trial (see Figure 2).

## Discussion

### Principal Findings

We aimed to examine participants’ experiences of using an attentive eating smartphone app, and we conducted exploratory analyses into the associations between usage of individual app functions and trial outcomes. Overall usage of app functions was reasonable (ie, total food entries, audio listens, and percentage of meals recorded using an image), except for total food gallery views, which was lower than expected: the median was less than one gallery view per day. Participants reported several barriers and facilitators to app use that could inform future research. Participants found that incorporating the use of the attentive eating app into one’s routine meant they were more likely to continue using it. This is in line with Ahtinen and colleagues [11], who found that once use of an app became routine, the app was easy to use. Future research should, therefore, include design of smartphone apps in such a way that facilitates their inclusion into a person’s routine; researchers may benefit from encouraging participants to use the app as part of their day-to-day habits early on in order to foster greater app usage throughout a study. The ability to access new and updated content has been found to motivate continued app usage [7,11], which is in line with our finding that boredom with the same audio clip may have led to reduced use of this function of the app. Further, believing that the app was effective was a motivator for continued use in this trial, as it was in another trial [11]. One way for future research studies to demonstrate app effectiveness to participants is to provide feedback on progress toward goals [7,11,29]. For example, feedback on weight loss would demonstrate app effectiveness to participants and likely bolster motivation to continue using the app. This study also found that some participants felt uncomfortable using the app in social situations. There was a notable age difference here, where younger participants were less likely to be concerned about being seen taking photographs of their food in social situations. Therefore, mobile phone apps to promote dietary change will benefit from being designed in ways that overcome concerns about their use in public. Further, some participants found the in-app rewards and badges motivating, whereas others did not. Future work should make such features optional or allow participants to personalize them.

Participants reported effects of using the app that were in line with the hypothesized mechanisms of action of the app. For example, participants reported using the food gallery to introspect on what they had eaten earlier and to inform subsequent food choices. Participants also described eating more slowly and stopping eating before finishing their food due to greater awareness of hunger and fullness, most likely due to the attentive eating audio clip that encouraged listeners to eat slowly and pay attention to their hunger and fullness levels. These findings are in contrast to the results of our main trial analyses, which found no effects on trial outcomes at the 8-week assessment, including weight and energy intake in the full sample and among a subsample of participants that were categorized as having used the app as intended [20]. Our exploratory analyses reported here found that the frequency of recording food consumed was significantly associated with lower body weight at the 8-week assessment but was not significantly associated with body fat percentage. This was a clinically relevant effect, with each additional food entry equating to 0.02 kg of weight loss. Recording four entries per day (ie, three meals and one snack) compared to recording only one entry per day would equate to an additional 3.6 kg of weight loss over a 2-month period. This amount of weight loss would be in line with recommendations on healthy weight loss of 0.5-1 kg per week [30]. We also found that the frequency of recording food consumed was significantly associated with greater self-reported 24-hour energy intake but not with taste-test energy intake. This was a small effect, with each additional food entry recorded equating to an additional 6 kcal consumed. Given the association with weight, we presume this may be a measurement artefact (ie, participants who diligently recorded meals in the app may have been more likely to diligently complete the dietary recalls).

Further examination of the number of food entries recorded over the entire trial period showed that while the number of food entries recorded was high at the beginning of the trial, it tended to decline over time. The decline in recording of food consumed may partially explain why the trial did not find any effects on main trial outcomes in the planned analyses. This finding is in line with previous work that found sustained use of technology-based interventions to be associated with better health outcomes [5]. More frequent entry of eating episodes allows for more frequent self-monitoring, and this may explain the association with weight, as self-monitoring is a known predictor of weight loss [31]. We found no association between how frequently participants viewed entries or listened to the attentive eating audio clip and weight, which may suggest that the act of recording behavior, as opposed to eating more attentively (ie, remembering food eaten and paying attention during eating), may explain why a greater number of eating episodes was associated with lower body weight. However, an alternative explanation is that more highly motivated participants were more likely to record their consumption episodes and were also more likely to lose weight.

### Strengths and Limitations

The use of qualitative research methods to examine participants’ experience of an attentive eating smartphone app is a strength of this work, as we have identified barriers and facilitators to using smartphone apps designed for dietary change and weight loss. Although our sample size was reasonable for qualitative analyses [32], it was small for our exploratory quantitative analyses and, for this reason, these results should be interpreted accordingly. Future confirmatory work is needed. A further limitation is that our measures of energy intake are prone to error and may, therefore, not accurately reflect energy intake during the trial, as one was self-reported and the other an objective laboratory measure of consumption of a single type of snack food.

### Conclusions

In conclusion, frequent recording of eating episodes using an attentive eating smartphone app was associated with greater weight loss during an 8-week randomized controlled trial. There are a number of barriers and facilitators to frequent use of an attentive eating mobile phone app that may be useful to address when designing dietary behavior change mobile phone apps.

## Abbreviations

CLAHRC: Collaboration for Leadership in Applied Health Research and Care
DALY: disability-adjusted life year
NIHR: National Institute for Health Research
VIF: variance inflation factor
